# Case report: Reversible encephalopathy caused by dyskinesia-hyperpyrexia syndrome

**DOI:** 10.3389/fneur.2023.1234974

**Published:** 2023-08-14

**Authors:** Bohan Luo, Hainan Zhang, Lixia Qin

**Affiliations:** Department of Neurology, The Second Xiangya Hospital, Central South University, Changsha, Hunan, China

**Keywords:** reversible encephalopathy, Parkinson's disease, dyskinesia-hyperpyrexia syndrome, emergency, MRI findings

## Abstract

Parkinson's disease (PD) is a common neurodegenerative disorder. Some patients with advanced-stage disease are accompanied by emergencies and critical issues such as dyskinesia-hyperpyrexia syndrome (DHS), parkinsonism-hyperpyrexia syndrome (PHS), and serotonin syndrome (SS). In this study, we report a patient with reversible encephalopathy caused by DHS who presented with an acute onset of fidgetiness, dyskinesia, and hyperpyrexia after antiparkinsonian drug abuse. In the present case, brain magnetic resonance imaging (MRI) showed multiple abnormal signals in the cortex and subcortex of the bilateral parietal and occipital lobes that resolved within weeks, which coincided with the characteristic MRI findings in posterior reversible encephalopathy (PRES). Our report expands on the neuroimaging features of DHS and highlights the importance of early identification, diagnosis, and treatment to improve patient prognosis.

## Introduction

Parkinson's disease (PD) is the second most common neurodegenerative disease and is clinically characterized by static tremors, bradykinesia, myotonia, and postural balance disorders (1). Critical and severe complications of PD occur mainly in advanced-stage patients and require urgent medical intervention. They are most commonly associated with worsening motor symptoms but can also manifest as severe non-motor complications. PD emergencies include dyskinesia-hyperpyrexia syndrome (DHS), parkinsonism-hyperpyrexia syndrome (PHS), levodopa-related motor complications, serotonin syndrome (SS), acute psychosis, and autonomic nervous system complications (2).

DHS is a rare but severe complication of PD. It is characterized by the acute onset of persistent systemic dyskinesia associated with increased creatine kinase (CK) levels, hyperpyrexia, and an altered mental state, and affects patients with advanced PD (2, 3). It typically occurs in patients with long disease durations undergoing high daily dopaminergic doses and can be triggered by changes in dopaminergic therapy, infections, hot weather, dehydration, or trauma (2, 4, 5). Although this rare acute complication has been reported in recent years, neuroimaging of DHS has been underreported. Herein, we report a rare case of reversible encephalopathy caused by DHS, emphasizing the neuroimaging findings.

## Case presentation

A 55-year-old man with a 10-year history of PD presented with an acute onset of consciousness disturbance and severe choreiform dyskinesia for 2 days. He had been diagnosed with PD 10 years earlier and showed a dramatic beneficial response to dopaminergic therapy. A pallidotomy had been performed 2 years earlier. No significant medical or family history was reported. The patient was treated for a long time with a combination of levodopa and benserazide (MADoPA), piribedil, selegiline, entacapone, and trihexyphenidyl. Only a few days prior, he increased the dosage of antiparkinsonian drugs on his own accord. The equivalent daily dose of levodopa was 1,750 mg per day. Two days earlier, he fell to the ground while going out on a hot afternoon. On admission, the patient exhibited confusion and continuous generalized involuntary movements without marked rigidity. Body temperature reached 40.6°C, and heart rate was 105 bpm. Systolic blood pressure was maintained at approximately 120–130 mmHg. The other neurological examination results were normal. Laboratory testing showed increased serum CK (1,468 U/L), CK-MB (38.8 U/L), and myoglobin (Mb) (374.2 μg/L). Other biochemical tests upon admission were negative, including alanine aminotransferase (130.1 U/L), aspartate aminotransferase (104.5 U/L), blood urea nitrogen (4.70 mmol/L), and creatinine (62.0 μmol/L).

**Figure 1:**
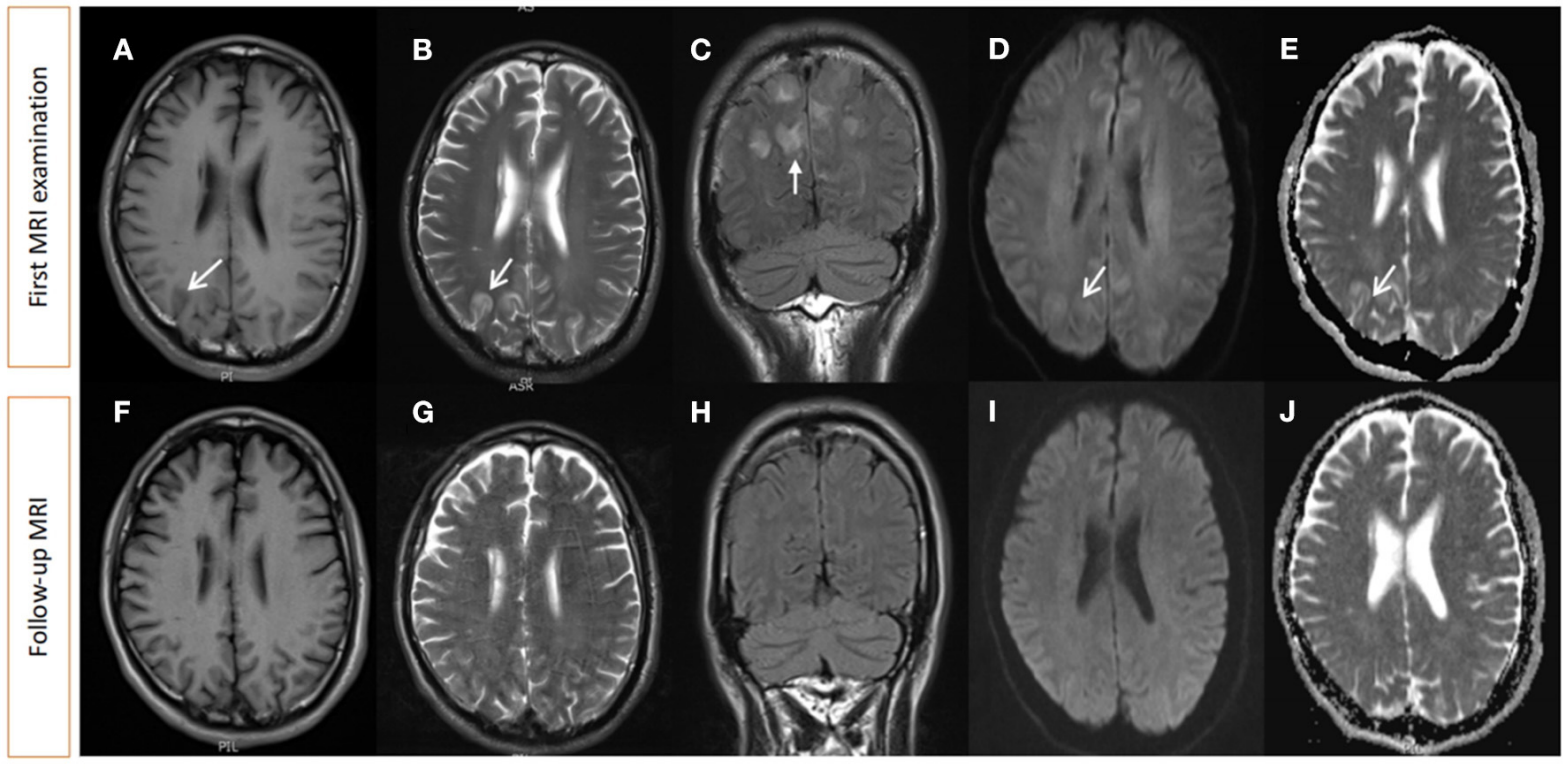
Brain MRI 19 days after symptom onset and at follow-up 3 months later. **(A)** T1 hypointensity and **(B)** T2 hyperintensity can be seen in the cortex and subcortex of the bilateral parietal lobe and the occipital lobe, with hyperintensity on T2 FLAIR **(C)**, DWI **(D)**, and ADC **(E)**. Follow-up MRI shows abnormalities have disappeared **(F–J)**. MRI, magnetic resonance imaging; FLAIR, fluid-attenuated inversion recovery; DWI, diffusion-weighted imaging; ADC, apparent diffusion coefficient.

Magnetic resonance imaging (MRI) performed 19 days after symptom onset revealed multiple abnormal signals of T1 and T2 hyperintensity in the cortex and subcortex of the bilateral parietal and occipital lobes, with hyperintensity on fluid-attenuated inversion recovery (FLAIR), diffusion-weighted imaging (DWI), and apparent diffusion coefficient (ADC), suggestive of prompt vasogenic edema (Figures 1A–E).

**Figure 2:**
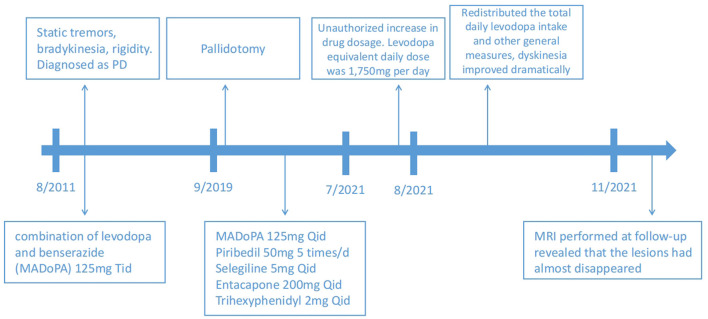
A timeline with relevant data from the episode of care.

Based on the patient's history and clinical presentation, a levodopa overdose was suspected. We immediately redistributed the total daily levodopa intake to 125 mg q. i.d., accompanied by amantadine hydrochloride 100 mg daily to control dyskinesia. Other general measures, such as rehydration and sedation, were used. After 5 days, dyskinesia improved dramatically, the CK level reduced to 529.6 U/L, and the CK-MB level reduced to 18.1 U/L as well. A further MRI performed at follow-up revealed that the lesions almost disappeared (Figures 1F–J). A time course of events can be found in Figure 2.

## Discussion

DHS, a rare but severe complication of PD, was first reported and defined as an emergency condition by Gil-Navarro et al. (6). DHS typically occurs in patients with advanced PD. Almost all patients have fluctuating symptoms and take high-dose dopaminergic drugs (2). It is often caused by the abuse of antiparkinsonian drugs. Several other triggering factors for DHS include infection, hot weather, dehydration, and trauma (2, 4, 5). Only a few cases have been reported thus far (4, 6–12) and few have described the neuroimaging features of DHS (4, 9).

Posterior reversible encephalopathy (PRES) is an acute or subacute onset reversible neurological disorder accompanied by various neurological symptoms such as headache, impaired visual acuity or visual field deficits, consciousness disturbances, seizures, encephalopathy, and focal neurological deficits (13, 14). The symptoms and signs of PRES are not specific; therefore, brain imaging is usually helpful in confirming the diagnosis of PRES. Characteristic MRI findings include bilateral regions of subcortical vasogenic edema that resolve within days or weeks (15). Our case coincided with MRI changes in PRES. This patient experienced consciousness disturbance, which can be classified as encephalopathy.

PRES often occurs due to abrupt blood pressure fluctuations, cytotoxic drugs, preeclampsia or eclampsia, sepsis, renal failure, or autoimmune diseases (13, 14). Cases of DHS leading to PRES have not been reported previously. We emphasize that DHS can cause reversible encephalopathy. However, the clinical manifestations of this patient did not conform to typical PRES. The patient did not have common clinical manifestations of PRES, such as elevated arterial pressure, seizures, and visual disturbances, and did not have the aforementioned causes; therefore, we speculate that the mechanism of DHS causing reversible encephalopathy in this patient may be related to direct endothelial injury reduced by hyperthermia, rhabdomyolysis, and high doses of antiparkinsonian drugs and not hypertension and cerebral hyperperfusion. Further studies are required to understand the underlying pathogenesis of this condition.

For the treatment of DHS, levodopa dosage should be gradually reduced. For patients with severe symptoms, appropriate sedation, fever reduction, hydration, and correction of electrolyte and acid-base imbalances can prevent renal failure, pneumonia, pulmonary embolism, and other complications. Most patients with DHS are relieved within a few days of active treatment and their prognosis is relatively good. PRES is generally benign. In many cases, PRES can be completely reversed within days to weeks of removing the triggering factors. After active treatment, the symptoms of DHS and PRES significantly improved. During follow-up, a head MRI showed that the original lesions had completely disappeared. Therefore, we believe that the prognosis of DHS-induced PRES caused by DHS is good.

## Conclusion

DHS is a rare, acute, treatable syndrome. Early blood biochemical tests (CK, CK-MB, etc.) and brain imaging tests are crucial for the timely and differential diagnosis of DHS. Head MRI may reveal bilateral asymmetric cortical and subcortical vasogenic edema, particularly in the parietal and occipital regions. This case report provides new insights into DHS and expands on its neuroimaging features.

### Patient perspective

“In 2011, I was diagnosed with PD and have been receiving oral anti-Parkinson's medication since then, with satisfactory symptom control. In July 2021, I felt that my symptoms were poorly controlled, so I increased my medication dosage on my own decision. A few days later, I suddenly experienced symptoms of severe involuntary movements and fell to the ground. My family sent me to the ICU for treatment. After the doctor helped me adjust my medication, my symptoms gradually recovered. After 2 weeks of treatment, I was discharged from the hospital and followed the doctor's instructions to take anti-Parkinson's drugs. No similar symptoms occurred again. Three months after discharge, I returned to the hospital for a re-examination of the head MRI, which showed that the lesion had almost disappeared.”

## Data availability statement

The raw data supporting the conclusions of this article will be made available by the authors, without undue reservation.

## Ethics statement

The studies involving human participants were reviewed and approved by the Ethics Committee of the Second Xiangya Hospital. The patients/participants provided their written informed consent to participate in this study. Written informed consent was obtained from the individual(s) for the publication of any potentially identifiable images or data included in this article.

## Author contributions

HZ contributed to the conception of the case report. BL conducted data organization and wrote the manuscript. LQ reviewed and revised the manuscript. All authors contributed to the article and approved the submitted version.
